# Perspectives of JAK Inhibitors for Large Vessel Vasculitis

**DOI:** 10.3389/fimmu.2022.881705

**Published:** 2022-03-30

**Authors:** Ryu Watanabe, Motomu Hashimoto

**Affiliations:** Department of Clinical Immunology, Osaka City University Graduate School of Medicine, Osaka, Japan

**Keywords:** giant cell arteritis, JAK inhibitors, large vessel vasculitis, Janus kinase (JAK), Takayasu arteritis

## Abstract

Vasculitis is an inflammation of the blood vessels caused by autoimmunity and/or autoinflammation, and recent advances in research have led to a better understanding of its pathogenesis. Glucocorticoids and cyclophosphamide have long been the standard of care. However, B-cell depletion therapy with rituximab has become available for treating antineutrophil cytoplasmic antibody-associated vasculitis (AAV). More recently, avacopan, an inhibitor of the complement 5a receptor, was shown to have high efficacy in remission induction against AAV. Thus, treatment options for AAV have been expanded. In contrast, in large vessel vasculitis (LVV), including giant cell arteritis and Takayasu arteritis, tocilizumab, an IL-6 receptor antagonist, was shown to be effective in suppressing relapse and has steroid-sparing effects. However, the relapse rate remains high, and other therapeutic options have long been awaited. In the last decade, Janus kinase (JAK) inhibitors have emerged as therapeutic options for rheumatoid arthritis (RA). Their efficacy has been proven in multiple studies; thus, JAK inhibitors are expected to be promising agents for treating other rheumatic diseases, including LVV. This mini-review briefly introduces the mechanism of action of JAK inhibitors and their efficacy in patients with RA. Then, the pathophysiology of LVV is updated, and a rationale for treating LVV with JAK inhibitors is provided with a brief introduction of our preliminary results using a mouse model. Finally, we discuss the newly raised safety concerns regarding JAK inhibitors and future perspectives for treating LVV.

## Introduction

Vasculitis syndrome is an autoimmune and/or autoinflammatory condition that causes inflammation of the blood vessels, and the resultant tissue ischemia causes damage to various organs. It is classified as large-, medium-, and small-vessel vasculitis according to the size of the affected blood vessels. The mainstay of treatment for vasculitis has long been glucocorticoids (GCs) and immunosuppressive agents such as cyclophosphamide, azathioprine, and methotrexate (MTX). However, new treatment options have long been awaited because of the significant burden of side effects of the treatment.

Treatment options for antineutrophil cytoplasmic antibody (ANCA)-associated vasculitis, particularly in microscopic polyangiitis and granulomatosis with polyangiitis, have expanded considerably in recent years. For example, B-cell depletion therapy using rituximab is as effective and safe as cyclophosphamide (1, 2). More recently, the efficacy and safety of avacopan, a complement 5a (C5a) receptor inhibitor that blocks neutrophil chemoattraction and activation, has been examined in ANCA-related vasculitis (3). This study showed that the C5a receptor blockade was superior to standard steroid therapy in remission induction at week 52, suggesting that avacopan may have the potential to replace standard steroid therapy (4).

In contrast, in large vessel vasculitis (LVV), including giant cell arteritis (GCA) and Takayasu arteritis (TAK), tocilizumab (TCZ), an IL-6 receptor antibody, is effective in preventing recurrence and reducing the dose of GCs (5, 6). However, the primary endpoint was not met in TAK (5), and many patients experienced relapse after discontinuation of TCZ (7), necessitating treatment that fundamentally improves vascular inflammation. Moreover, blockade of T cell costimulation signals using abatacept is effective and safe in GCA (8), but failed to show its efficacy in TAK (9). TAK often affects young women, and the side effects of accumulated steroids owing to multiple relapses are immense (10). Many of the drugs used in real-world clinical practice for TAK lack sufficient evidence in randomized controlled trials (11). Thus, unmet clinical needs remain for LVV, particularly in TAK.

In the last 10 years, Janus kinase (JAK) inhibitors have emerged as promising agents for rheumatology (12). JAK inhibitors are low-molecular-weight compounds that can be orally administered to patients with rheumatoid arthritis (RA), unlike biological disease-modifying antirheumatic drugs (bDMARDs) (13). Their efficacy and safety have been compared with those of bDMARDs and have been proven in multiple studies in patients with RA.

This mini-review briefly explains the mechanism of action of JAK inhibitors and their efficacy in patients with RA. Then, we update the pathophysiology and provide a rationale for treating LVV with JAK inhibitors. Finally, we discuss the safety and future perspectives of JAK inhibitors for LVV treatment.

## Success of JAK Inhibitors in RA

### Mechanism of Action

**Figure 1:**
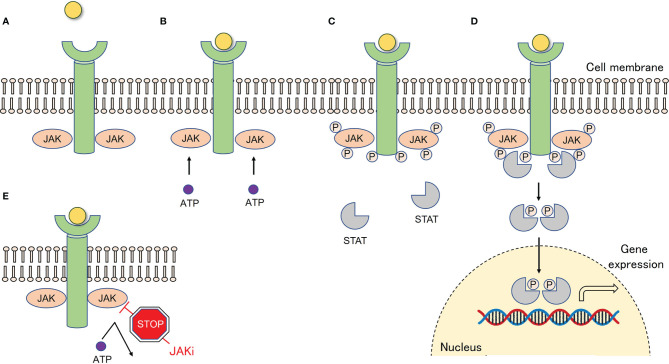
The JAK-STAT pathway and mechanism of action of JAK inhibitors. **(A)** Type I and type II cytokines utilize the Janus kinase (JAK)-signal transducer and activation of transcription (STAT) pathway. **(B)** When type I and II cytokines bind to their receptors on the cell surface, JAKs bound to the intracellular domains are phosphorylated by adenosine triphosphate binding. **(C)** Phosphorylated JAKs, in turn, phosphorylate the receptor end. **(D)** The transcription factor STAT binds to the receptor end, and phosphorylated STATs form a dimer, which is then transferred to the nucleus to regulate gene expression. **(E)** JAK inhibitors competitively bind to the binding site of ATP, inhibiting phosphorylation of JAK and exerting their effects. ATP, Adenosine triphosphate; JAK, Janus kinase; JAKi, Janus kinase inhibitor; P, Phosphate; STAT, Signal transducer and activator of transcription.

Cytokine receptors are grouped into several superfamilies based on their shared structural elements of the receptors (14). Type I and type II cytokines utilize the JAK-signal transducer and activation of transcription (STAT) pathway **(** **Figure 1** **)**. When type I and II cytokines bind to their receptors on the cell surface, JAKs bound to the intracellular domains are phosphorylated by adenosine triphosphate binding, which in turn phosphorylates the receptor end. The transcription factor STAT binds to the receptor end, and phosphorylated STATs form a dimer, which is then transferred to the nucleus to regulate gene expression (15). There are four isoforms of JAKs (JAK1, JAK2, JAK3, and TYK2). Type I and type II cytokines include the common γ chain family (IL-2, 4, 7, 9, 13, and 15), gp130 cytokines (IL-6, Oncostatin M), granulocyte colony-stimulating factor (G-CSF), granulocyte macrophage colony-stimulating factor (GM-CSF), interferon (IFN)-α, β, γ, IL-12, and others, but not tumor necrosis factor α (TNF-α), IL-1, IL-17, and TGF-β (12).

### Efficacy of JAK Inhibitors in RA

The efficacy of JAK inhibitors has been tested in head-to-head comparisons with adalimumab, a representative TNF-α inhibitor, in multiple trials involving patients with RA. The results demonstrated that JAK inhibitors are non-inferior or even superior to adalimumab in controlling disease activity (16–18). Based on these results, JAK inhibitors have been placed equal to bDMARDs in the most updated RA treatment recommendations (19). In other words, when methotrexate fails to induce remission, RA patients can choose either bDMARDs or JAK inhibitors. Thus, JAK inhibitors are an essential therapeutic option for the treatment of RA.

## JAK Inhibitors for Vasculitis

### Large Vessel Vasculitis: GCA and TAK

Both GCA and TAK affect the aorta and its major branches and are characterized by granulomatous vascular inflammation (20). IFN-γ and IL-17 derived from Th1 and Th17 cells are the dominant cytokines (21–23), and neoangiogenesis or new formation of vasa vasorum in the adventitia and lumen occlusion due to intimal hyperplasia can be observed in both diseases (24). Although many disease mechanisms are shared, several differences exist. For example, granulomatous lesions mainly contain CD4^+^ T cells and macrophages in GCA, whereas CD8^+^ T and NK cells are also involved in TAK (25). In the peripheral blood, the follicular helper T cell-B cell signature, which promotes immunoglobulin production, is highly enriched in TAK, but not in GCA (26). Adventitial fibrosis is more prominent in TAK than in GCA (25). Thus, from a pathomechanistic point of view, TAK is more complex than GCA and a single therapeutic target alone may not be sufficient to achieve remission. In this context, JAK inhibitors are expected to be effective because of the simultaneous blockade of multiple cytokines, especially in TAK.

Our previous work showed enhanced activity of the JAK-STAT pathway in the vascular lesions of patients with GCA (27). Compared with biopsy-negative temporal arteries, biopsy-positive temporal arteries showed elevated transcripts of STAT1, STAT2, and STAT4, as well as target genes corresponding to each STAT. Moreover, cytokine production in CD4^+^ T cells from patients with GCA was dependent on the JAK-STAT pathway, as demonstrated by an experiment showing that tofacitinib, an inhibitor of JAK1 and JAK3, inhibited IFN-γ production in a dose-dependent manner (27). In line with this report, a recent study from a French group demonstrated that STAT1 and STAT2 transcripts were highly upregulated in aortic lesions of GCA by using microarray analysis (28). In addition, both CD4^+^ and CD8^+^ T cells in the peripheral blood of patients with GCA showed increased activity of the JAK-STAT pathway. The same group also identified upregulated JAK-STAT signals in both CD4^+^ and CD8^+^ T cells in the peripheral blood of patients with TAK (29).

**Figure 2:**
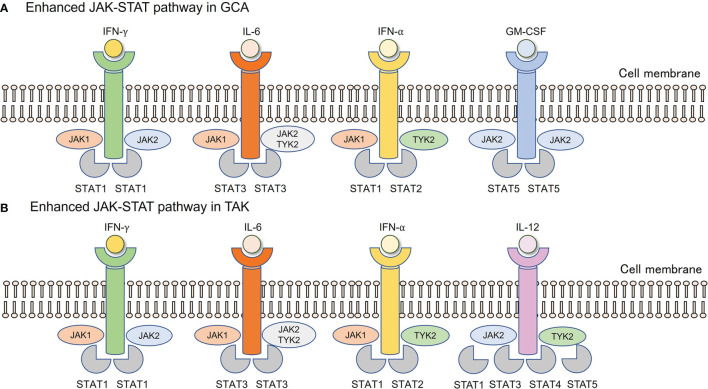
Enhanced JAK-STAT pathway in GCA and TAK. **(A)** In vascular lesions of GCA, IFN-γ derived from Th1 cells, IL-6 from tissue macrophages, IFN-α, and GM-CSF derived from unknown origin are enriched. **(B)** In vascular lesions of TAK, IFN-γ derived from Th1 cells, IL-6 from tissue macrophages, IFN-α from unknown origin, and IL-12 from monocyte/macrophages are enriched. Each cytokine utilizes its JAKs, followed by corresponding STAT phosphorylation. GCA, Giant cell arteritis; GM-CSF, Granulocyte macrophage colony-stimulating factor; IFN, Interferon; JAK, Janus kinase; STAT, Signal transducer and activation of transcription; TAK, Takayasu arteritis.

What is the mechanism underlying the enhanced activity of the JAK-STAT pathway in LVV **(** **Figure 2** **)**? This question is equivalent to asking which type I and II cytokines are implicated in GCA and TAK. Undoubtedly, IL-6 plays a key role in both diseases, as suggested by the clinical effects of TCZ (5, 6). IL-6 is mainly derived from CD68^+^ tissue macrophages in both GCA and TAK (30, 31), and IL-6 primarily utilizes STAT3 as a downstream transcription factor (32). Since the above-mentioned studies have demonstrated that STAT3 is highly activated in CD4^+^ and CD8^+^ T cells in both diseases (28, 29), this IL-6-STAT3 axis substantially contributes to the pathogenesis of both diseases. However, this axis alone does not explain the increased activity of STAT1 and STAT2 signals in the vascular lesions of the GCA (27, 28).

In recent years, type I IFNs have attracted attention in the pathophysiology of LVV. Upregulation of type I IFNs, particularly IFN-α, has been reported in the serum, temporal arteries, and aortic lesions of GCA (27, 28). A highly enriched type I IFN signature has been observed in both CD4^+^ and CD8^+^ T cells from TAK patients (29). The binding of type I IFNs to their receptors activates JAK1 and TYK2, which is followed by the phosphorylation of STAT1 and STAT2 (33), which fits perfectly into the context of what has been reported so far. Although plasmacytoid dendritic cells are the main source of type I IFNs in systemic lupus erythematosus, those in GCA and TAK remain unknown (34). In addition to type I IFNs, the role of GM-CSF in the promotion of vascular inflammation in GCA has been reported (35). Thus, IL-6, type I IFNs, and GM-CSF are involved in the pathogenesis, all of which utilize the JAK-STAT pathway, making it highly likely that JAK inhibitors are effective against GCA.

Furthermore, a genome-wide association study identified *IL-12B* as a susceptibility gene for TAK (36, 37). Serum IL-12 levels are elevated in TAK patients (38), and the risk allele of *IL-12B* is closely associated with vascular damage in TAK (39). IL-12 uses the JAK-STAT pathway, and JAK2 and TYK2 are located in the downstream signaling pathway (40). Thus, JAK inhibitors are expected to be effective against TAK (41).

Based on these findings, we examined the effects of tofacitinib, which blocks JAK1 and JAK3, on LVV in a mouse model (27). In this mouse model, human medium-sized arteries were engrafted into immunodeficient mice, and vascular inflammation was induced by injecting lipopolysaccharide and peripheral blood mononuclear cells from patients with GCA. In this model, tofacitinib not only inhibited T-cell activation and cytokine production but also inhibited macrophage activation, resulting in the efficient suppression of vascular inflammation. Analysis of T cells in vasculitic lesions identified a highly proliferative population, called “tissue-resident memory T (Trm) cells”. Trm cells express CD69 and CD103 and show a rapid response to antigens once encountered. These cells may have the potential to induce a relapse of vascular inflammation in GCA (42). Our results demonstrate that these cells can be targeted by tofacitinib as well (27).

In line with these data from basic research, several case reports describing the efficacy of JAK inhibitors on LVV have been published (29, 43–48). Very recently, baricitinib, an inhibitor of JAK1 and JAK2, was reported to be effective against relapsing GCA in a prospective open-label study (49). Although the number of enrolled patients was small, the high remission induction and steroid withdrawal rates suggest that this treatment is promising for GCA. In addition, the efficacy and safety of tofacitinib and MTX were prospectively evaluated in active Takayasu arteritis (50). Compared to MTX-treated group, complete remission and steroid reduction rates were higher in the tofacitinib-treated group, but relapse and imaging improvement rates did not reach the statistical significance. Other clinical trials of JAK inhibitors for GCA (NCT03725202, upadacitinib) and TAK (NCT04161898, upadacitinib) are ongoing. TAK may be less likely to produce good results than GCA because of the complexity of the disease mechanism; however, we are awaiting promising results.

### Other Forms of Vasculitis

Once the efficacy of JAK inhibitors has been experimentally demonstrated in LVV, they are expected to be effective in other forms of vasculitis. Some pilot studies and case reports demonstrated the efficacy of JAK inhibitors for ANCA-associated vasculitis (51), polyarteritis nodosa (52), cutaneous leukocytoclastic vasculitis (53), and vascular Behcet’s disease (54); however, data on other forms of vasculitis are very limited (55), and we cannot get any conclusion from such limited data.

## Discussion

So far, we have focused on the efficacy of JAK inhibitors for rheumatic diseases. As for safety, data are accumulating on the treatment of RA. The use of JAK inhibitors is associated with a higher risk of developing shingles, reactivation of varicella-zoster virus (VZV), than bDMARDs (56). An increased risk of serious infections compared to bDMARDs has also been reported in some trials (57). In addition, new safety concerns emerged after the results of an Oral Surveillance trial were published (58). In this trial, patients with active RA who were at risk for cardiovascular events, such as smoking, were assigned to one of three treatment groups: TNF inhibitors, or 5 mg of tofacitinib twice daily, or 10 mg twice daily, and observed for 5 years. The results showed an increased risk of death, malignancy, major adverse cardiac events (MACE), and venous thromboembolism (VTE) in tofacitinib-treated patients (both 5mg and 10 mg arms) compared to those treated with TNF inhibitors (58). In September 2021, the Food and Drug Administration issued a warning regarding the use of JAK inhibitors. Subsequently, the use of JAK inhibitors for patients with RA is, in principle, limited to patients who are refractory to at least one TNF inhibitor. Although selection bias, which only recruited patients at risk of cardiovascular events, cannot be ruled out in the study, and real-word data from a large cohort do not support the increased risk of such serious adverse events (59), we agree that screening before administration and regular monitoring during administration are essential for the treatment with JAK inhibitors.

GCA patients are often older than RA patients and are at higher risk of serious infection, MACE, and VTE (60–62). Therefore, it is recommended that JAK inhibitors be administered only after adequate risk management and cardiovascular prevention. With regard to shingles, it has been reported that VZV is a contributing factor in the development of GCA (63, 64) and is considered extremely high-risk in elderly patients with GCA. In the study of baricitinib for GCA described above, it was reported that the live-attenuated zoster vaccine did not prevent the onset of shingles (49). It has been reported that recombinant adjuvanted zoster vaccine can suppress the onset of herpes zoster at a high rate in RA patients (65). Therefore, administration of this recombinant vaccine prior to the use of JAK inhibitors is desirable in patients with GCA.

In conclusion, the efficacy of JAK inhibitors in treating rheumatic diseases is promising. Given their pathophysiology, JAK inhibitors should have high efficacy for GCA and TAK. Therefore, the results of these clinical trials are awaited. However, new safety concerns have emerged that may be limited to treatment-resistant cases. There is an urgent need to establish the long-term safety of JAK inhibitors.

## Author Contributions

RW drafted the manuscript. MH revised and finalized the manuscript. All authors contributed to the article and approved the submitted version.

## Funding

This work was in part supported by JSPS KAKENHI Grant Number 20K17418 and a grant-in-aid of the Cardiovascular Research Fund, Tokyo, Japan to RW.

## Conflict of Interest

RW receives speaker’s fee from Eli Lilly. MH receives research grants from AbbVie, Asahi-Kasei, Brystol-Meyers, Eisai, Eli Lilly, Novartis Pharma.

## Publisher’s Note

All claims expressed in this article are solely those of the authors and do not necessarily represent those of their affiliated organizations, or those of the publisher, the editors and the reviewers. Any product that may be evaluated in this article, or claim that may be made by its manufacturer, is not guaranteed or endorsed by the publisher.
